# When algorithms set the roster: algorithmic scheduling, work-time control, and work-related burnout among hotel employees

**DOI:** 10.3389/fpsyg.2026.1958975

**Published:** 2026-10-07

**Authors:** Mingzhuo Wang, Puvaneswaran Kunasekaran

**Affiliations:** 1 School of Business, Qingdao University, Qingdao, Shandong, China; 2 Faculty of Human Ecology, Universiti Putra Malaysia, Selangor, Malaysia

**Keywords:** algorithmic management, algorithmic scheduling, burnout, family-supportive supervisor behaviors, work–family conflict, work–home resources model, work-time control

## Abstract

Hotels increasingly use algorithms to align staffing with fluctuating demand, yet little is known about how algorithmic scheduling is associated with employees’ work–family strain. This study applies the work–home resources model to examine how algorithmic scheduling exposure is associated with hotel employees’ work-related burnout through low work-time control and work–family conflict and whether family-supportive supervisor behaviors (FSSB) attenuate this resource-loss sequence. A cross-sectional, self-report survey was conducted among frontline hotel employees in Shandong Province, China, from May to June 2026. Data from respondents were analyzed using partial least squares structural equation modelling with 10,000 bootstrap subsamples. Algorithmic scheduling exposure was positively associated with low work-time control and work–family conflict. Low work-time control partly mediated the scheduling–conflict association; work–family conflict was positively associated with burnout; and the serial indirect association was significant. FSSB weakened both the scheduling–control association and the serial indirect association with burnout, although the former remained significant at high FSSB. These associations remained significant, but several were attenuated after controlling for measured working time and family circumstances. The findings distinguish an algorithmic management practice from the temporal resource employees experience under it and show how the associated resource loss crosses the work–family boundary. By locating FSSB at the algorithm–employee interface, the study conceptualizes hotel scheduling as hybrid temporal governance: algorithms allocate working time, while supportive supervision is associated with a weaker exposure–control relationship. Hotels should therefore evaluate scheduling systems by the stability, advance notice, and negotiability of the working time they produce, rather than by operational efficiency alone.

## 1 Introduction

Time is a core resource in hotel work. Hotels need to allocate personnel according to fluctuating demand at night, weekends and holidays, and algorithmic scheduling can integrate demand forecasts and labor supply information, and incorporate employees’ availability or personal preferences in systems, so as to quickly complete this task (Parent-Rocheleau et al., 2024). Hotel scheduling is situated within broader changes in employment and workplace governance. Tourism industries employ more than 270 million people worldwide, while persistent problems of variable working hours and insecure employment make working-time arrangements a significant social concern (Sodergren, 2024). Algorithmic management is reshaping employment relations across industries by changing how organizations direct, evaluate, and discipline workers (Kellogg et al., 2020). Its growing integration into hotel operations similarly changes frontline managers’ responsibilities, requiring them to coordinate algorithmic systems while supporting employees (Jianu et al., 2025a). In fact, in the tourism and hospitality industry, more predictable work arrangements are associated with higher employee satisfaction (Yang et al., 2026). However, automation may also shift the decision-making over working hours from employees and responsible managers to computational rules. Different from the schedules formulated by the manager, when the optimization logic of the algorithm lacks transparency, and its output is presented as the result of a technically determined decision, the scheduling generated by the algorithm may be more difficult to negotiate and adjust (Parent-Rocheleau and Parker, 2022; Jianu et al., 2025a). The algorithm may also divide labor into finer time units and extend the time interval during which employees must remain available for work at any time (Piasna, 2024). Therefore, the algorithmic scheduling itself is not necessarily harmful. Its core theoretical significance is that it may reallocate the control of time when work occurs. The key issue is not only whether the hotel uses the algorithm, but how algorithmic scheduling is transformed into employees’ control of working hours.

The existing two streams of research can only partially answer this question. Research on workplace technology in the hospitality industry focuses on general AI awareness, service robots, job insecurity, and employees’ reactions to future automation (Koo et al., 2021; Bakir et al., 2025). Although AI awareness is relevant, its conceptual scope is broader than algorithmic management. Algorithmic management itself includes different functions such as monitoring, goal setting, scheduling, performance evaluation and compensation management (Parent-Rocheleau et al., 2024). Recent hospitality research has revealed the implementation of algorithmic management, humanization, and other issues, but these systems are generally considered as a comprehensive management configuration for investigation, without distinguishing the scheduling function separately (Jianu et al., 2025a, 2025b, 2025c). On the other hand, work–family research shows that irregular working hours, variable scheduling and low working-time control are closely related to work–family conflict and strain, which is particularly obvious in the hospitality industry (McNamara et al., 2011; Xu and Cao, 2019; O’Neill and Follmer, 2020; Cho et al., 2024). Relevant studies also found that the impact of AI awareness or algorithmic control may cross the work–family boundary (Zheng and Zhang, 2025; Chen and Chen, 2026; Yi and Kumar, 2026), but these studies did not examine the specific function of directly allocating employees’ working time. It can be seen that the existing studies either discuss algorithmic management without specifically distinguishing the scheduling function, or focus on scheduling quality without identifying its algorithmic source. Therefore, it is still not fully understood how algorithmic scheduling can be transformed into work–family strain through the working-time control that employees actually feel.

Distinguishing between perceived exposure to algorithmic scheduling and low working-time control can help to bridge the gap between the above studies. The former reflects the extent to which the algorithmic system determines or affects employees’ schedules, but does not evaluate whether the system is fair, transparent or useful (Parent-Rocheleau et al., 2024). The latter is a situational work resource, which is defined in this paper as unpredictable working hours per week, short-notice schedule changes, and the difficulty for employees to obtain the requested leave time (McNamara et al., 2011). The work–home resources model provides the overall theoretical framework for this study and incorporates the resource-loss logic emphasized by conservation of resources theory (ten Brummelhuis and Bakker, 2012; Hobfoll et al., 2018). According to the model, when the intervention of the algorithm in scheduling weakens the control of employees’ working time, this intervention constitutes a contextual demand in the domain of work. The proposed explanation is that limited work-time control constrains personal resources such as time and energy, making family-role performance more difficult and linking work–family conflict with burnout (Xu and Cao, 2019).

A hybrid temporal governance perspective recognizes that algorithms and human managers may jointly influence scheduling. Algorithms can generate schedules, while front-line managers may interpret, implement, or modify their output where they retain the authority to do so. The practice of managers in specific situations can make algorithmic management more enabling or more coercive (Jianu et al., 2025a, 2025b). Family-supportive supervisor behaviors (FSSB) play an important role in this specific translation process. FSSB includes emotional support, role modelling, instrumental support, and creative management of work–family demands (Hammer et al., 2013). Where supervisors have relevant authority, assistance with shift exchanges or family needs could make schedules more adjustable. Evidence from the catering and hospitality industries shows that FSSB is associated with lower strain levels and better work–family outcomes (Jolly et al., 2022; Ye et al., 2024). Although FSSB is a broader concept, the first-stage moderation proposed in this study is mainly based on the specific actions that the supervisor has the authority to take. Therefore, this research model compares two explanations about algorithmic management: one view is that automation replaces the discretion of managers; another view is that the algorithm and the supervisor jointly participate in the management of employee time.

This study examines how exposure to algorithmic scheduling is associated with work-related burnout through low working-time control and work–family conflict among hotel employees. Meanwhile, this study also tests whether FSSB weaken the relationship between algorithmic scheduling and working-time control. This study mainly makes three contributions. First, it shifts the focus of hotel employee–technology research from general AI awareness to the specific management function of algorithmic scheduling. Second, by distinguishing between algorithmic scheduling exposure and employees’ actual perception of working-time control, this study explains how technological governance can be translated into resource loss, and then cross the work–family boundary and become related to burnout. Third, it conceptualizes hotel scheduling as hybrid temporal governance: algorithms generate time allocations, whereas supportive supervision may condition employees’ experienced work-time control. Placing FSSB at the interface between the algorithm and employees helps to identify a human mechanism through which the hotel can maintain a certain degree of employees’ control over working hours in the algorithmically managed scheduling system.

## 2 Literature review and hypothesis development

### 2.1 The work–home resources perspective

The work–home resources (W–HR) model provides the theoretical framework for this study. Conservation of resources (COR) theory holds that people seek to retain and protect valued resources. Its loss-primacy principle gives resource loss greater weight than comparable gains, while resource investment helps prevent or recover from loss. The W–HR model applies this logic to the work–home interface: contextual demands can deplete personal resources and thereby impair functioning in the other domain (ten Brummelhuis and Bakker, 2012; Hobfoll et al., 2018). In contrast, contextual resources help individuals protect or generate personal resources such as time, physical strength, attention and positive emotions. This resource-gain route explains enrichment, whereas the loss route explains conflict. Contextual resources are features of the environment, such as schedule control or supervisor support; personal resources, such as energy and attention, reside in the individual. Resource availability and contextual conditions may therefore qualify cross-domain associations. When work demands consume resources needed for family roles, work–family conflict is expected (Greenhaus and Beutell, 1985; ten Brummelhuis and Bakker, 2012). This study focuses on the direction of work affecting family, and defines work–family conflict as the degree to which work requirements hinder individuals from fulfilling family responsibilities (Netemeyer et al., 1996).

The W–HR model is the organizing framework, with COR providing its underlying resource-loss logic rather than a separate parallel explanation. Here, algorithmic scheduling exposure is a perceived work arrangement that may be demanding when associated with constraints on employees’ time. Work-time control is a contextual work resource; a high low-control score indicates limited access to that resource, not an observed loss over time. FSSB is a contextual social resource that may qualify the exposure–control association. Work–family conflict represents cross-domain interference, while burnout represents work-related strain. Time and energy depletion provide a theoretical link but were not measured, and the study does not test enrichment or reciprocal home-to-work processes. Consequently, the cross-sectional paths assess associations consistent with the proposed resource account, not its temporal sequence. Figure 1 presents the model.

**Figure 1:**
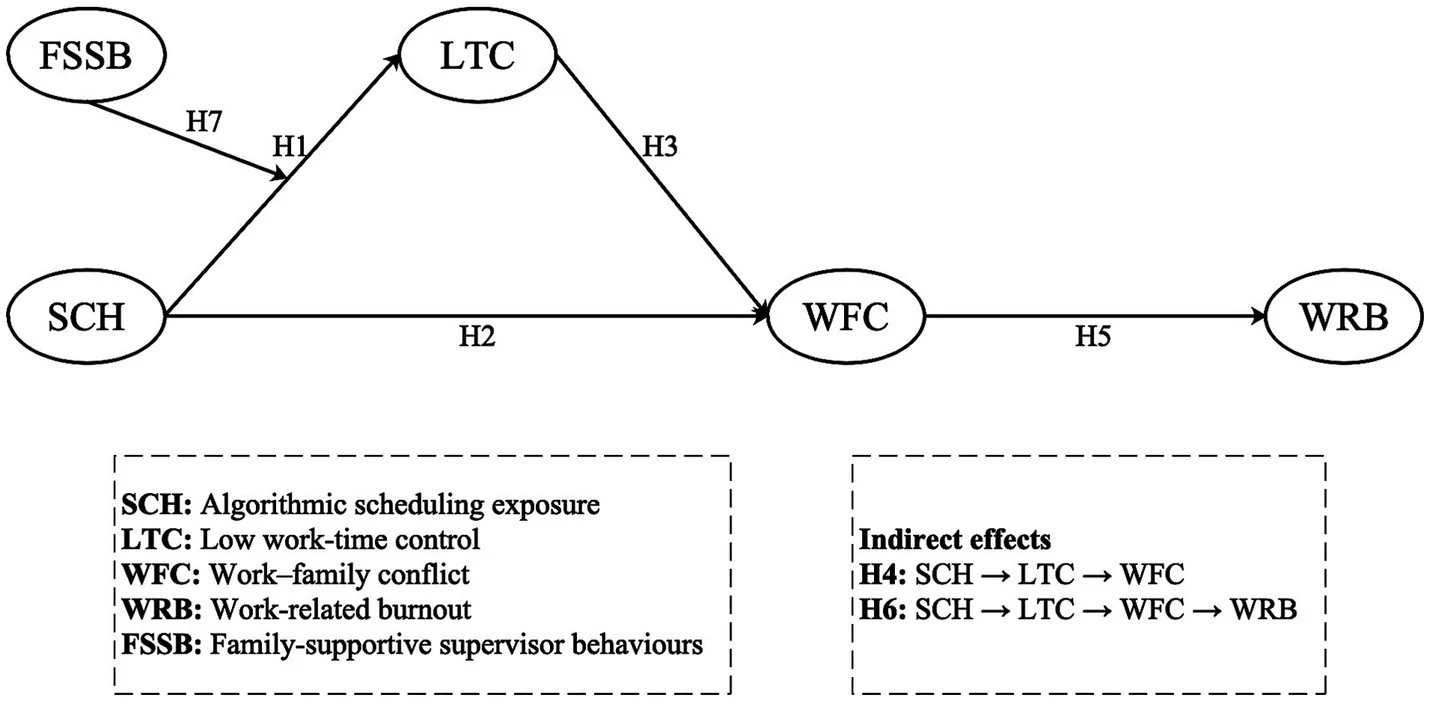
Conceptual framework. The figure presents the focal hypotheses. Estimation includes the FSSB main effect on LTC and the SCH × FSSB interaction; H8 is the index of the conditional serial indirect association. The adjusted sensitivity model adds the control paths specified in Data analysis.

### 2.2 Algorithmic scheduling exposure and low work-time control

Algorithmic management includes different functions such as monitoring, goal setting, performance evaluation, compensation and scheduling (Parent-Rocheleau et al., 2024). In this study, algorithmic scheduling exposure refers to employees’ perceived extent of automated-system involvement in deciding when and for how long they work and in preparing their schedules. It is not a technical audit of the system or a measure of algorithm quality. The scheduling algorithm can integrate demand forecast, staffing level, employee availability, performance data and other operational information, and allocate or adjust shifts with limited manual intervention (Parent-Rocheleau and Parker, 2022). This function should be distinguished from general AI awareness, because it directly governs the way employees access working time and non-working time.

When consistency, fairness and employee preferences are included in the system design, algorithmic scheduling may improve the consistency of scheduling, reduce the bias of managers, and better take care of employee preferences. Therefore, its impact is not predetermined by the technology itself. Process-quality features are possible explanations or boundary conditions, but they are not captured by algorithmic scheduling exposure and were not tested in this study. However, greater reliance on automated scheduling may shift influence over working-time decisions away from employees, providing a resource-based rationale for an association between exposure and low work-time control (Parent-Rocheleau and Parker, 2022; Jianu et al., 2025a). Algorithmic management may compress the expression space of employees in work design, while automatic scheduling may produce rapid and frequent adjustments, making it difficult for employees to question or renegotiate relevant arrangements (Parent-Rocheleau and Parker, 2022; Piasna, 2024). Recent hospitality research also shows that the consequences of algorithmic management depend on whether employees and managers can understand, modify or question the algorithm output (Jianu et al., 2025b).

The low work-time control in this paper refers to the extent to which employees believe that they cannot affect their work-time arrangement, which is specifically manifested in difficulty in predicting weekly working hours, short-notice schedule changes, and difficulty in obtaining requested time off (McNamara et al., 2011). It is a kind of working condition that employees actually experience, rather than a synonymous expression of whether scheduling technology exists or not. It is important to distinguish between the two: the algorithmic system determines the source and intensity of scheduling intervention, and low work-time control reflects how this intervention is transformed into the temporal experience of employees. As automated systems assume a greater role in allocating working time, employees’ ability to coordinate work and family may depend more on the scheduling arrangements they experience. This study tests the exposure–control association, without identifying opacity or non-negotiability as its mechanism. Based on this, this study proposes the following hypothesis:

*H1*: Algorithmic scheduling exposure is positively associated with low work-time control.

### 2.3 Algorithmic scheduling exposure and work–family conflict

Low work-time control cannot exhaust all the ways in which algorithmic scheduling enters the family life of employees. The concept of temporal intrusion in a broader sense reflects how work takes up the time that employees would otherwise reserve for family, rest or personal activities. The algorithm can divide work into finer time units and extend the time that employees must remain available for work, even if this waiting time is not formally included in the schedule (Piasna, 2024). Demand-responsive systems may also generate non-social working hours, weekly work-hour fluctuations, short-notice notifications, or pressure to accept work arrangements at short notice.

Unpredictable working hours, limited advance notice, and last-minute schedule changes may obstruct employees from arranging child care, family affairs and other scheduled responsibilities, thus aggravating the conflict between work and family (Henly and Lambert, 2014; Cho et al., 2024). Similar conclusions have been drawn from the research on algorithmically mediated work. A two-wave study on gig workers found that algorithmic observational monitoring was positively correlated with work–family conflict because workers needed to invest time and attention to deal with irregular system requirements (Chen and Chen, 2026). The national survey evidence further shows that platform work is related to a higher degree of work–family conflict, and work contact outside working hours can explain part of the relationship (Glavin et al., 2024). More direct evidence from the hospitality industry shows that weekly ICT availability requirements extended beyond formal working hours reduced the performance of employees’ family roles by weakening their psychological detachment (Zhang et al., 2026).

The W–HR model interprets the above phenomenon as a process of resource depletion: short-notice work demands, undesirable working hours and continued availability conditions may consume employees’ time, attention and energy, and these resources cannot be put into family roles at the same time. Even if employees can retain certain influence on individual shifts, as long as the scheduling system causes variable in working hours, or keeps them digitally tethered to potential scheduling adjustments, employees may still experience work–family conflicts. Based on this, this study proposes the following hypothesis:

*H2*: Algorithmic scheduling exposure is positively associated with work–family conflict.

### 2.4 Low work-time control, work–family conflict, and mediation

The control of working time enables employees to align shifts with periodic family responsibilities, plan care, protect shared time, and recover between different working periods. Therefore, work-time control is a contextual resource, not just the absence of work demands (ten Brummelhuis and Bakker, 2012). When work-time control is low, employees have to repeatedly invest time and cognitive effort to coordinate the conflict between assigned working hours and family responsibilities. Some conflicts are difficult to resolve through re-coordination, resulting in time-based conflicts. Meanwhile, the pressure caused by scheduling uncertainty and coordination failure may also continue to affect employees’ ability to participate in family life after work (Greenhaus and Beutell, 1985).

Hospitality industry research and the broader work–family research provide consistent evidence. Among hotel employees, unpredictable working hours, short-notice schedule changes and difficulty in obtaining time off were all related to higher levels of work–life conflict (McNamara et al., 2011). A systematic review covering 53 studies concluded that work-time control was associated with better work–non-work balance (Nijp et al., 2012). More powerful evidence comes from an organizational intervention study. The intervention improved employees’ control over working time and place, and then significantly reduced their work–family conflict (Kelly et al., 2014). At the industry level, a meta-analysis of the hospitality industry found that job control was negatively related with work–non-work conflict (Xu and Cao, 2019). These findings support the basic argument of the resource perspective, that is, time control helps employees protect the resources they need in the family domain.

*H3*: Low work-time control is positively associated with work–family conflict.

H1 and H3 together describe a transformation mechanism. Algorithmic scheduling is a governance practice, and low work-time control reflects the remaining temporal autonomy experienced by employees under this governance practice. Distinguishing these two constructs helps to explain how a technological arrangement translates into cross-domain resource depletion. In view of H2’s argument that algorithmic scheduling may also cause additional temporal intrusion through scheduling time, work-hour variability and availability expectations, low work-time control is expected to only transfer part of the relationship between algorithmic scheduling and work–family conflict, rather than necessarily explain the whole relationship. Based on this, this study proposes the following hypothesis:

*H4*: Low work-time control plays a partial mediating role in the positive relationship between algorithmic scheduling exposure and work–family conflict.

### 2.5 Work–family conflict, work-related burnout, and serial mediation

Work-related burnout refers to the long-term physical and psychological exhaustion that employees attribute to their work (Kristensen et al., 2005). The reason that work–family conflict can lead to such depletion is that the interference of work on family life will prevent employees from replenishing and restoring resources in non-work domains. The time spent dealing with work–family conflicts will reduce the opportunities for employees to rest and participate in family life, while concerns about the failure to fulfill family responsibilities will keep employees in a state of continuous cognitive and emotional activation after work. Therefore, recurrent work–family conflict may gradually transform the immediate cross-domain contradiction into cumulative work-related strain.

Meta-analysis evidence shows that work–family conflict is closely related to emotional exhaustion, psychological distress and other indicators of impaired well-being (Amstad et al., 2011). This association is particularly relevant in hospitality industry, because irregular working hours and continuous service requirements make it more difficult for employees to fully recover. A meta-analysis of the hospitality industry found that there was a strong positive relationship between work–non-work conflict and burnout (Xu and Cao, 2019). Hospitality empirical research also shows that work–family conflict is positively correlated with emotional exhaustion, and emotional exhaustion will further transfer its negative impact to job performance and other work outcomes (Karatepe, 2013; Wang et al., 2021). These findings are consistent with the W–HR model: when work continues to consume the resources needed by employees in the family domain, it is more difficult for employees to restore the energy required for subsequent work.

*H5*: Work–family conflict is positively associated with work-related burnout.

The above hypotheses together constitute a theoretically ordered resource depletion process. Algorithmic scheduling may weaken a contextual work resource, that is, employees’ control over working time. Lower work-time control will further consume personal resources needed by employees to coordinate their family roles, thus aggravating work–family conflict; recurring work–family conflict will limit the recovery of employees and is associated with work-related burnout. Based on this, this study proposes the following hypothesis:

*H6*: Algorithmic scheduling exposure has a positive serial indirect association with work-related burnout through low work-time control and work–family conflict.

### 2.6 The moderating role of FSSB and hybrid temporal governance

FSSB refers to the actions taken by supervisors to help employees coordinate work and family responsibilities. This construct comprises emotional support, instrumental support, role modelling, and creative work–family management (Hammer et al., 2009, 2013). This kind of specificity is very important. A meta-analysis found that work–family-specific support was more closely related to work–family conflict than general supervisor support (Kossek et al., 2011). Intervention research also shows that FSSB can be improved through supervisor training (Hammer et al., 2011). In hospitality industry, FSSB is associated with lower emotional exhaustion and more positive work–family outcomes among hospitality employees (Jolly et al., 2022; Ye et al., 2024).

In this research model, FSSB is not positioned as a general buffer resource after work–family conflict, but acts at the interface between algorithmic allocation and employees’ actual perception of work-time control. Hotel scheduling is a kind of hybrid temporal governance: the algorithm may generate time allocation schemes, but the supervisor is still responsible for explaining, implementing, recalibrating or overriding these schemes. Research on hotel algorithmic management regards front-line managers as intermediaries and algorithmic coaches between the algorithmic system and employees, who are responsible for translating system output, incorporating specific situational knowledge and promoting employees’ expression of opinions (Jianu et al., 2025a). Relevant hospitality research also shows that whether the algorithmic system is more humanized depends on how managers and employees interpret, negotiate and question the algorithm output (Jianu et al., 2025b).

The supportive supervisor may approve employees to change shifts, adjust system parameters, consider employees’ family constraints, or raise objections to unreasonable time allocation. Emotional support and role modelling can enhance the legitimacy of such needs, but pure sympathy cannot really change the scheduling. Therefore, when the supervisor still retains actual discretion in the process of scheduling, FSSB should be able to weaken the transformation from algorithmic intervention to low work-time control. At a higher level of FSSB, the output of the algorithm still has room for negotiation and adjustment; however, at a lower level of FSSB, the same algorithm output is more likely to be regarded as a fixed and unchangeable arrangement. Based on this, this study proposes the following hypothesis:

*H7*: FSSB weakens the positive association between algorithmic scheduling exposure and low work-time control.

If FSSB can protect employees’ work-time control in the first stage of the resource loss process, its impact should further extend to the entire indirect chain. A higher level of FSSB should weaken the correlation between algorithmic scheduling and low work-time control, and thus weaken the correlation between algorithmic scheduling and work–family conflict and work-related burnout. Based on this, this study proposes the following hypothesis:

*H8*: The positive serial indirect association between algorithmic scheduling exposure and work-related burnout through low work-time control and work–family conflict will weaken with the increase of FSSB level.

## 3 Materials and methods

### 3.1 Measurement

Algorithmic scheduling exposure was measured by the four-item scheduling scale in the Algorithmic Management Questionnaire developed by Parent-Rocheleau et al. (2024). The scale mainly assesses the extent to which the automated system determines when employees work, prepares the work schedule, and determines working hours. It therefore measures perceived exposure/intensity only, not process quality such as transparency, fairness, employee input, or negotiability. A higher score does not indicate that the system is more opaque, unfair, or rigid. The four items map to employees’ perceived allocation of scheduling decisions to an automated system; they do not identify its vendor, architecture, data inputs, optimization objective, or decision rules. No technical system audit was conducted. All items were scored using the seven-point Likert scale, where 1 means “completely disagree” and 7 means “completely agree.” This study adjusted the guiding example of platform work in the original scale to the hotel operation situation, but retained the substantive content of the items.

Low work-time control was measured with the three-item scale used by McNamara et al. (2011) in the study of hotel employees. Respondents need to report how often they have experienced the following situations: unpredictable working hours per week, short-notice shift changes, and failure to obtain the requested time off. Each item is scored with a five-point frequency scale, where 1 means “never” and 5 means “always.” Therefore, the higher the score, the lower the employee’s control over working time. Work–family conflict was measured with the five-item work-to-family scale developed by Netemeyer et al. (1996). The scale reflects the interference of work requirements, working hours, and work pressure, and is scored with the seven-point Likert scale, in which 1 means “strongly disagree” and 7 means “strongly agree.”

Work-related burnout was measured using the seven-item work-related burnout subscale of the Copenhagen Burnout Inventory (Kristensen et al., 2005). The scale mainly assesses the exhaustion and frustration that employees think are caused by work. According to the design of the original scale, the answer options of frequency items are from “always” to “never/almost never,” and the degree items are from “very high” to “very low.” According to the scoring method of the original scale, all the answers are converted to 0–100 points. The higher the score, the higher the degree of work-related burnout of employees. Family-supportive supervisor behaviors (FSSB) were measured with the four-item scale developed by Hammer et al. (2013). The four items represent emotional support, instrumental support, role modelling and creative work–family management, and are scored with the five-point Likert scale, in which 1 means “strongly disagree” and 5 means “strongly agree.”

For the adjusted analysis, the questionnaire supplied average actual weekly working hours, main shift pattern, relationship status, number of children under 18, and current responsibility for caring for a child or dependent adult. Hours and child counts entered as numeric variables. Mainly night work was coded 1 and other main shift patterns 0; this indicator does not measure night-shift frequency or identify occasional night work among rotating-shift employees. Caregiving was coded 1 for yes and 0 for no. Reported married/cohabiting status was coded 1 (*n* = 396), with the remaining responses coded 0 (*n* = 239). Weekly hours include total work time but do not separately identify overtime.

The English scales were translated into simplified Chinese by a combination of blind translation and back translation (Brislin, 1970). First of all, two bilingual researchers completed the Chinese translation independently and formed a unified translation through discussion. Subsequently, another bilingual researcher who had not been exposed to the original English scales translated the unified Chinese version back into English. An expert panel composed of two scholars with backgrounds in hospitality management and organizational behavior, respectively, evaluated the semantic equivalence, situational applicability and item clarity of the Chinese and English versions. After that, this study conducted cognitive interviews with 15 front-line hotel employees to identify expressions that may have ambiguity, especially focusing on the comprehensibility of the definition of automated scheduling system. On this basis, 100 eligible employees were pre-tested. The modification after the pilot test is limited to improving the clarity of the item expression, and no item is added or deleted before the formal investigation. See Appendix 1 for all measurement items.

### 3.2 Data collection

The study population of this study is the front-line employees of three-star to five-star hotels in Shandong Province, China. This study collected data through a one-time anonymous questionnaire from May to June 2026. Twelve candidate hotels were identified through existing university–industry partnerships with the researchers’ college. The study uses purposive sampling to recruit respondents through participating hotels. The research team contacted hotel management to explain the study and seek permission to invite employees. Hotels that agreed to facilitate access circulated the survey invitation among their frontline employees. Hotel participation enabled access to potential respondents, while individual participation remained voluntary. Neither hotels nor employees were selected through probability sampling. Respondents were eligible if they were at least 18 years old, worked in a frontline or operational hotel role, had at least 3 months’ tenure at their current hotel, and had experienced a schedule that was at least partly generated, revised, or influenced by an automated or electronic system during the previous 3 months. The sample comprised front office (*n* = 141), housekeeping (149), food and beverage (160), kitchen (79), banquets/events (61), and other departments (45). Departmental composition is reported in Table 1; departmental differences in scheduling demand were not separately modeled. The first page of the questionnaire describes the purpose of the study, the voluntary nature of participation, the right of respondents to stop answering at any time before submitting the questionnaire, and the confidentiality measures used to protect confidentiality. All respondents began to fill in the questionnaire after reading the relevant instructions and providing informed consent.

**Table 1:** Demographic statistics of respondents.

Variable	Scale	Frequency	Percentage
Age	18–24 years	121	19.06%
	25–34 years	256	40.31%
	35–44 years	197	31.02%
	45 years or older	61	9.61%
Gender	Woman	389	61.26%
	Man	246	38.74%
Relationship status	Single	219	34.49%
	Married/cohabiting	396	62.36%
	Separated/divorced	11	1.73%
	Widowed	1	0.16%
	Prefer not to answer	8	1.26%
Education	High school or below	160	25.20%
	College diploma	222	34.96%
	Bachelor’s degree	213	33.54%
	Postgraduate degree	34	5.35%
	Other	6	0.94%
Department	Front office	141	22.20%
	Housekeeping	149	23.46%
	Food and beverage	160	25.20%
	Kitchen	79	12.44%
	Banquets/events	61	9.61%
	Other	45	7.09%

*A priori* statistical power analysis was completed by G*Power. The results showed that at least 184 samples were needed to detect the coefficient of determination (R^2^) of no less than 0.10 when the significance level was 5% and the statistical power was 95% (Hair et al., 2022). The final sample size of this study is higher than the minimum requirement. Of the total of 710 questionnaires, 635 were valid and thus retained after data screening, with a usable completion rate of 89%. Screening focused on completion times shorter than 5 min and clearly repetitive or patterned responding, such as systematic selection of fixed response categories. The demographic characteristics of the sample are shown in Table 1.

### 3.3 Data analysis

This study used SmartPLS 4 software and partial least squares structural equation modelling (PLS-SEM) to estimate the research model (Ringle et al., 2024). Since this study adopts a single-wave and self-report survey design, the study controls common method bias (CMV) procedurally and statistically. Procedural control measures include anonymity, the use of neutral instructions, the separation of construct blocks, the non-disclosure of the hypothesized model, the use of clear and non-evaluative wording, and the retention of the response format of each original scale. In terms of statistical test, this study adopted two CMV test methods suitable for PLS-SEM, namely the correlation matrix procedure (Bagozzi et al., 1991) and the full collinearity test (Kock, 2015). According to the correlation matrix procedure, CMV usually does not pose a serious problem when the correlation coefficients between various constructs are lower than 0.90. Using Pearson product–moment correlations among the five unweighted construct mean scores, the maximum absolute correlation coefficient is 0.492 (Table 2), which is between work–family conflict and work-related burnout, and is significantly lower than the threshold of 0.90. For the full collinearity test, Kock (2015) suggested that the variance inflation factor (VIF) should not exceed 3.30. The full collinearity VIFs of the five constructs are between 1.128 and 1.668. Therefore, both test results showed that CMV was unlikely to have a substantial impact on the results of this study.

**Table 2:** Means, standard deviations, and pearson correlations.

Construct	Mean	SD	1	2	3	4	5
1. SCH	4.234	1.653	—				
2. LTC	2.980	1.086	0.369	—			
3. WFC	4.243	1.628	0.391	0.418	—		
4. WRB	50.017	26.803	0.175	0.166	0.492	—	
5. FSSB	3.020	1.092	−0.092	−0.333	−0.100	−0.049	—

*N* = 635. Mean and SD are based on unweighted item means; SD uses *N* − 1. Pearson correlations are shown below the diagonal. Scale ranges are 1–7 for SCH and WFC, 1–5 for LTC and FSSB, and 0–100 for WRB. SCH = algorithmic scheduling exposure; LTC = low work-time control; WFC = work–family conflict; WRB = work-related burnout; FSSB = family-supportive supervisor behaviors.

To assess sensitivity to measured working time and family circumstances, one adjusted model was estimated alongside the unadjusted model. Weekly hours and mainly night work were linked to LTC, WFC, and WRB; reported married/cohabiting status, children under 18, and caregiving were linked to WFC and WRB, adding 12 control paths. The first group represents working-time demands potentially shared by exposure and strain; the second represents family circumstances relevant to conflict and burnout. Both models retain the FSSB main effect and SCH × FSSB interaction predicting LTC. They use the same 635 cases, indicators, and 10,000-subsample, two-tailed BCa bootstrap procedure. It does not isolate a causal effect: working hours may themselves depend on scheduling, and several alternative explanations remain unmeasured. No hotel-level clustering adjustment was possible because respondent-to-hotel identifiers were not retained and cannot be recovered.

## 4 Results

Table 2 reports means, sample standard deviations, and Pearson product–moment correlations for the five main constructs. Descriptive scores are unweighted means of the items in their analysis-ready direction, with work-related burnout expressed on the 0–100 scale. These descriptive scores are distinct from the latent scores used in the PLS-SEM estimation.

### 4.1 Measurement model assessment

This study first evaluates the reflective measurement model. According to the general specifications of PLS-SEM results reporting, the evaluation include indicator reliability, internal consistency reliability, convergent validity and discriminant validity (Ali et al., 2018; Hair et al., 2019, 2022). As shown in Table 3, the standardized outer loadings of all measurement items are between 0.769 and 0.906, which is higher than the recommended standard of 0.708. Cronbach’s α was between 0.804 and 0.923, ρA was between 0.806 and 0.925, and composite reliability was between 0.884 and 0.938, all in the acceptable range of 0.70–0.95. The average variance extracted (AVE) of each construct ranged from 0.686 to 0.767, which were higher than the threshold of 0.50, indicating that the measurement model had good convergent validity.

**Table 3:** Measurement model assessment.

Construct	Indicator	Loading	α	ρA	ρC	AVE
Algorithmic scheduling exposure (SCH)	SCH1	0.875	0.899	0.900	0.929	0.767
	SCH2	0.906				
	SCH3	0.867				
	SCH4	0.854				
Low work-time control (LTC)	LTC1	0.866	0.804	0.806	0.884	0.718
	LTC2	0.840				
	LTC3	0.836				
Work–family conflict (WFC)	WFC1	0.884	0.913	0.917	0.935	0.743
	WFC2	0.888				
	WFC3	0.865				
	WFC4	0.844				
	WFC5	0.827				
Work-related burnout (WRB)	WRB1	0.801	0.923	0.925	0.938	0.686
	WRB2	0.835				
	WRB3	0.802				
	WRB4	0.769				
	WRB5	0.859				
	WRB6	0.839				
	WRB7	0.886				
Family-supportive supervisor behaviors (FSSB)	FSSB1	0.859	0.867	0.872	0.909	0.715
	FSSB2	0.847				
	FSSB3	0.863				
	FSSB4	0.811				

α = Cronbach’s alpha; ρA = reliability coefficient; ρC = composite reliability; AVE = average variance extracted.

This study used the heterotrait–monotrait ratio (HTMT) to evaluate discriminant validity. Compared with the Fornell–Larcker criterion or cross-loadings, HTMT is considered to be a more appropriate method for testing discriminant validity (Henseler et al., 2015). The HTMT estimates of each construct ranged from 0.076 to 0.536, which were lower than the conservative 0.85 criterion. In addition, all the 95% bias-corrected and accelerated (BCa) bootstrap confidence intervals do not include 1. The above results show that the five constructs of this study have good discriminant validity.

### 4.2 Structural model assessment

The following results and Tables 3–7 describe the unadjusted model. The adjusted comparison is reported in Table 8. This study evaluated the structural model for predictor collinearity, explanatory power of the model, and effect size (Hair et al., 2019, 2022). The inner variance inflation factor (VIF) of the structural model is between 1.000 and 1.158, which is significantly lower than the conservative critical value of 3, indicating that there is no obvious collinearity issue between the predictors. The model explained 24.1% variation of low work-time control, 24.2% variation of work–family conflict, and 24.3% variation of work-related burnout.

**Table 4:** Heterotrait–monotrait ratios.

Construct	SCH	LTC	WFC	WRB	FSSB
SCH					
LTC	0.434 [0.353, 0.510]				
WFC	0.432 [0.356, 0.501]	0.488 [0.415, 0.555]			
WRB	0.192 [0.109, 0.273]	0.192 [0.109, 0.278]	0.536 [0.467, 0.597]		
FSSB	0.104 [0.048, 0.186]	0.399 [0.316, 0.476]	0.112 [0.056, 0.192]	0.076 [0.043, 0.114]	

Values in square brackets are 95% bias-corrected and accelerated bootstrap confidence intervals.

**Table 5:** Structural model assessment.

Endogenous construct	Predictor	Inner VIF	*f* ^2^	*R* ^2^	Adjusted *R*^2^
LTC	SCH	1.010	0.149	0.241	0.238
	FSSB	1.010	0.122		
	FSSB × SCH	1.001	0.018		
WFC	SCH	1.158	0.087	0.242	0.240
	LTC	1.158	0.116		
WRB	WFC	1.000	0.322	0.243	0.242

General *f*^2^ benchmarks are 0.02, 0.15, and 0.35 for small, medium, and large effects; interaction-specific benchmarks are 0.005, 0.010, and 0.025, respectively (Kenny, 2018).

**Table 6:** Hypothesis testing.

Hypothesis	Relationship	*β*	SE	*t*	*p*	95% BCa CI	Supported
H1	SCH → LTC	0.338	0.034	9.929	< 0.001	[0.268, 0.401]	Yes
H2	SCH → WFC	0.276	0.037	7.367	< 0.001	[0.200, 0.346]	Yes
H3	LTC → WFC	0.318	0.036	8.918	< 0.001	[0.246, 0.386]	Yes
H4	SCH → LTC → WFC	0.107	0.016	6.655	< 0.001	[0.078, 0.141]	Yes
H5	WFC → WRB	0.493	0.031	15.750	< 0.001	[0.429, 0.551]	Yes
H6	SCH → LTC → WFC → WRB	0.053	0.009	5.985	< 0.001	[0.037, 0.072]	Yes
H7	FSSB × SCH → LTC	−0.116	0.034	3.455	< 0.001	[−0.181, −0.049]	Yes
H8	FSSB × SCH → LTC → WFC → WRB	−0.018	0.006	3.118	0.002	[−0.031, −0.008]	Yes

Two-tailed inference is based on 10,000 bootstrap subsamples. BCa CI = bias-corrected and accelerated confidence interval.

**Table 7:** Conditional effects at levels of family-supportive supervisor behaviors.

Effect	FSSB level	*β*	SE	*t*	*p*	95% BCa CI
Panel A. conditional SCH → LTC association						
SCH → LTC	Low (−1 SD)	0.453	0.043	10.633	< 0.001	[0.365, 0.532]
SCH → LTC	Mean	0.338	0.034	9.929	< 0.001	[0.268, 0.401]
SCH → LTC	High (+1 SD)	0.222	0.052	4.236	< 0.001	[0.116, 0.324]
Panel B. conditional serial indirect association with WRB						
SCH → LTC → WFC → WRB	Low (−1 SD)	0.071	0.012	6.135	< 0.001	[0.050, 0.096]
SCH → LTC → WFC → WRB	Mean	0.053	0.009	5.985	< 0.001	[0.037, 0.072]
SCH → LTC → WFC → WRB	High (+1 SD)	0.035	0.010	3.666	< 0.001	[0.018, 0.055]

Low and high FSSB represent one standard deviation below and above the mean, respectively. Two-tailed inference is based on 10,000 bootstrap subsamples. BCa CI = bias-corrected and accelerated confidence interval.

**Table 8:** Comparison of focal associations before and after adjustment.

Hypothesis or association	Base *β*	Base 95% BCa CI	Adjusted *β*	Adjusted 95% BCa CI	Adjusted *p*
H1 SCH → LTC	0.338	[0.268, 0.401]	0.248	[0.179, 0.314]	< 0.001
H2 SCH → WFC	0.276	[0.200, 0.346]	0.224	[0.153, 0.293]	< 0.001
H3 LTC → WFC	0.318	[0.246, 0.386]	0.195	[0.121, 0.266]	< 0.001
H4 SCH → LTC → WFC	0.107	[0.078, 0.141]	0.048	[0.028, 0.073]	< 0.001
H5 WFC → WRB	0.493	[0.429, 0.551]	0.483	[0.409, 0.550]	< 0.001
H6 SCH → LTC → WFC → WRB	0.053	[0.037, 0.072]	0.023	[0.013, 0.037]	< 0.001
H7 SCH × FSSB → LTC	−0.116	[−0.181, −0.049]	−0.114	[−0.173, −0.052]	< 0.001
H8 FSSB × SCH → LTC → WFC → WRB	−0.018	[−0.031, −0.008]	−0.011	[−0.019, −0.005]	0.003

*N* = 635 in both models. All base-model *p* values are < 0.001 except H8 (*p* = 0.002). Estimates and 95% BCa intervals use 10,000 individual-level bootstrap subsamples. Weekly hours and mainly night work predict LTC, WFC, and WRB; reported married/cohabiting status, children under 18, and caregiving predict WFC and WRB.

The result of the effect size *f*^2^ shows that the contribution of work–family conflict to work-related burnout is the largest in the model (*f*^2^ = 0.322), followed by the impact of algorithmic scheduling exposure on low work-time control (*f*^2^ = 0.149). The *f*^2^ of other main effects ranged from 0.087 to 0.149. The effect size of the interaction term was 0.018. This result is classified as medium using interaction-specific *f*^2^ reference values of 0.005, 0.010, and 0.025 for small, medium, and large effects, respectively (Kenny, 2018). See Table 5 for complete results of the structural model.

### 4.3 Hypothesis testing and conditional process effects

This study uses 10,000 bootstrap subsamples, two-tailed tests and 95% BCa confidence intervals to test the research hypothesis. As shown in Table 6, the results showed that algorithmic scheduling exposure was significantly positively associated with low work-time control [*β* = 0.338, *p* < 0.001, 95% BCa CI (0.268, 0.401)], and H1 was supported. After low work-time control was incorporated into the model, there was still a significant positive relationship between algorithmic scheduling exposure and work–family conflict (*β* = 0.276, *p* < 0.001), and H2 was supported. There was also a significant positive relationship between low work-time control and work–family conflict (*β* = 0.318, *p* < 0.001), and H3 was supported.

The specific indirect relationship between algorithmic scheduling exposure and work–family conflict through low work-time control was significant [*β* = 0.107, *p* < 0.001, 95% BCa CI (0.078, 0.141)]. Since the indirect relationship and the remaining direct relationship are both positive and reach a significant level, the mediation model represents complementary partial mediation (Nitzl et al., 2016), and H4 is supported. Further analysis found that work–family conflict was significantly positively associated with work-related burnout [*β* = 0.493, *p* < 0.001, 95% BCa CI (0.429, 0.551)], and H5 was supported. The serial indirect relationship between algorithmic scheduling exposure and work-related burnout through low work-time control and work–family conflict was also significant [*β* = 0.053, *p* < 0.001, 95% BCa CI (0.037, 0.072)], and H6 was supported.

In general, the two modeled routes together form a significant total indirect relationship between algorithmic scheduling exposure and work-related burnout [*β* = 0.189, *p* < 0.001, 95% BCa CI (0.148, 0.231)]. The total indirect relationship can be further divided into two parts: one is through the serial path of low work-time control and work–family conflict in turn (*β* = 0.053); the second is only through the indirect path of work–family conflict (*β* = 0.136). This decomposition result is consistent with the theoretical distinction of this research model, that is, on the one hand, algorithmic scheduling may lead to resource loss by weakening employees’ work-time control; on the other hand, temporal intrusion may still exist after controlling for work-time control.

The interaction between FSSB and algorithmic scheduling exposure was negative and significant [*β* = −0.116, *p* < 0.001, 95% BCa CI (−0.181, −0.049)], and H7 was supported. Simple slope analysis showed that when FSSB was at a low level, the positive relationship between algorithmic scheduling exposure and low work-time control was the strongest (*β* = 0.453); when FSSB was at the mean level, the relationship was weakened (*β* = 0.338); when FSSB is at a high level, the relationship is the weakest (*β* = 0.222). The relationship under the three conditions reached a significant level, but the slope showed a systematic decline, indicating a weaker exposure–control association at higher FSSB, rather than directly demonstrating improved scheduling negotiability. See Table 7 and Figure 2 for the specific results.

**Figure 2:**
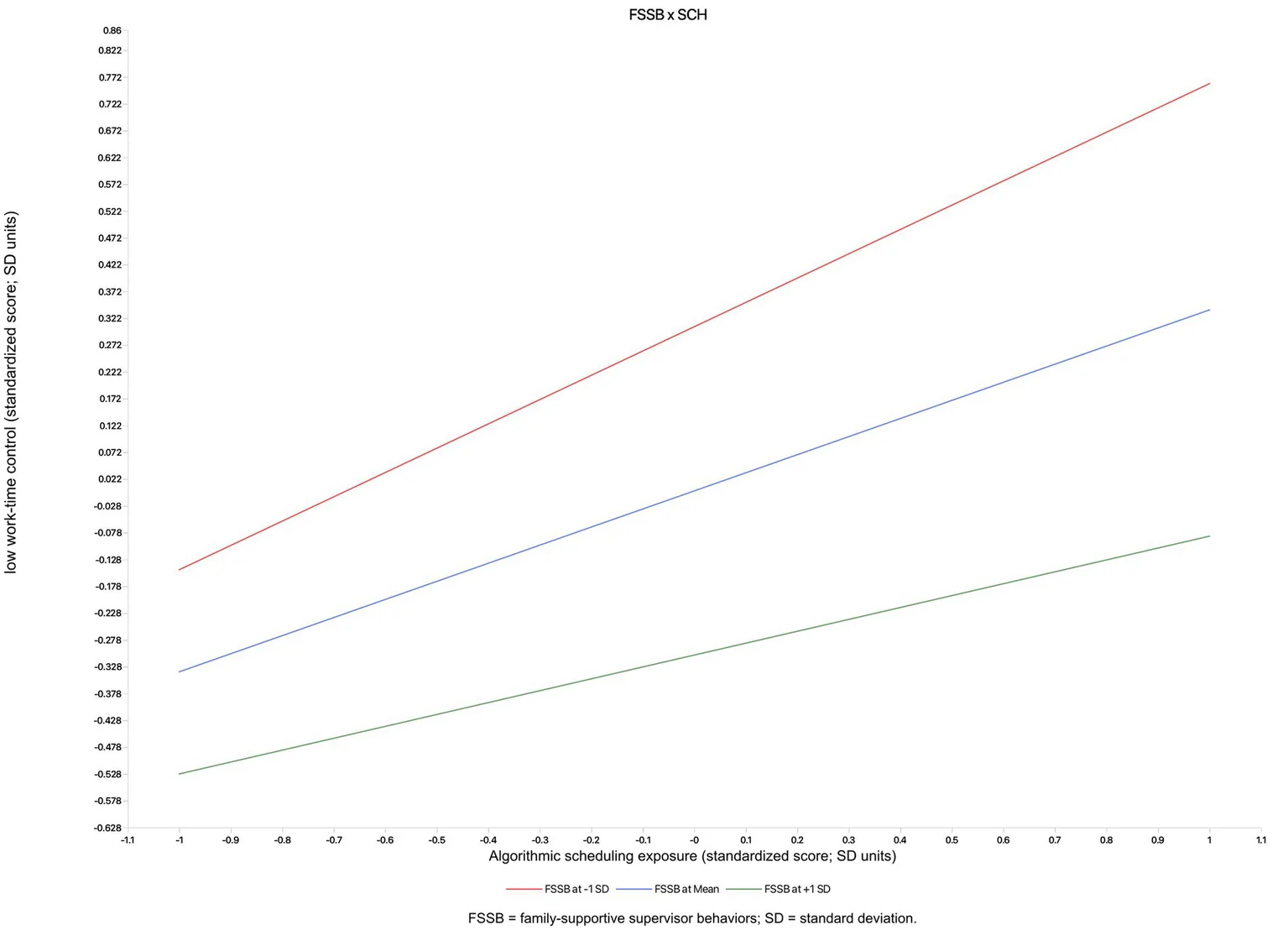
Moderating effect of family-supportive supervisor behaviors.

Finally, the index of moderated serial mediation, namely the specific indirect relationship of the interaction term through low work-time control and work–family conflict, is negative and significant [*β* = −0.018, *p* = 0.002, 95% BCa CI (−0.031, −0.008)], and H8 is supported. The conditional serial indirect relationship gradually weakens with the increase of FSSB level: *β* = 0.071 at low FSSB level, *β* = 0.053 at mean FSSB level, and *β* = 0.035 at high FSSB level, and the BCa confidence intervals under the three conditions do not include 0. It can be seen that the higher level of FSSB weakens the complete process of resource loss between algorithmic scheduling exposure and work-related burnout.

### 4.4 Sensitivity analysis with measured controls

Table 8 compares the unadjusted and adjusted estimates. All eight focal associations retained their directions and their 95% BCa intervals excluded zero. However, several estimates were attenuated: SCH → LTC decreased from 0.338 to 0.248, SCH → WFC from 0.276 to 0.224, and LTC → WFC from 0.318 to 0.195. The serial indirect association decreased from 0.053 to.023 [95% BCa CI (0.013, 0.037)], indicating appreciable sensitivity in magnitude to the included covariates rather than unchanged results.

The interaction remained negative [*β* = −0.114, *p* < 0.001, 95% BCa CI (−0.173, −0.052)], as did the estimates of moderated serial mediation [*β* = −0.011, *p* = 0.003, 95% BCa CI (−0.019, −0.005)]. Adjusted SCH–LTC slopes were 0.362 at low FSSB and 0.134 at high FSSB; the corresponding serial indirect estimates were 0.034 at low FSSB and 0.013 at high FSSB.

## 5 Discussion and conclusion

This study examines how algorithmic scheduling exposure is associated with work-related burnout of frontline and operational hotel employees, and under what conditions this association will be weakened. The results are consistent with the theoretically specified pattern of associations, rather than establishing a resource-loss process. A higher degree of algorithmic scheduling exposure is associated with lower work-time control and higher work–family conflict; low work-time control partially mediate the relationship between algorithmic scheduling exposure and work–family conflict; work–family conflict has a strong positive correlation with work-related burnout. FSSB not only weakened the first-stage relationship between algorithmic scheduling exposure and low work-time control, but also weakened the complete serial indirect relationship between algorithmic scheduling exposure and work-related burnout. The findings link algorithmic scheduling exposure with employees’ experienced work-time control and identify FSSB as a moderator. Whether supervisors achieved this through negotiation or schedule adjustment was not directly tested. The adjusted analysis retained these associations but substantially reduced the serial indirect estimate, indicating that measured working time and family circumstances share explanatory relevance with the proposed account.

The positive relationship between algorithmic scheduling exposure and low work-time control is consistent with the view that algorithms may reshape work by reallocating autonomy and management authority (Parent-Rocheleau and Parker, 2022; Piasna, 2024). This finding also complements the conclusion of recent hospitality research that algorithmic constraints and algorithmic opacity may reduce the well-being of employees, and human intervention can make the algorithmic system more enabling (Jianu et al., 2025b, 2025c). However, the present findings do not test opacity, rigidity, fairness, or negotiability as mechanisms, because these process-quality attributes were not measured. The results of this study do not imply that automated scheduling is inherently harmful, but show that the wider involvement of algorithms is related to a specific temporal experience, which is manifested in the difficulty of predicting working hours, short-notice schedule changes and difficulty in obtaining the requested time off. By distinguishing the two, this study goes beyond the general attitude of employees towards AI and identifies an employee resource that may be affected by specific management functions.

The research results of low work-time control and work–family conflict are consistent with the existing scheduling research, but also expand it. Among Australian hotel employees, in addition to long working hours and high work intensity, lower hours control is also related to a higher level of work–life conflict (McNamara et al., 2011). National survey evidence also shows that the fluctuation of working hours will aggravate the conflict between work and family, and when the fluctuation of working hours is large, employees’ substantive control over working hours can play a protective role (Cho et al., 2024). Organizational intervention research further proves that improving employees’ control over working hours and places can reduce work–family conflict (Kelly et al., 2014).

Low work-time control only partially mediates the relationship between algorithmic scheduling exposure and work–family conflict. The remaining direct relationship has important theoretical significance, because it is consistent with temporal intrusion beyond the ability of employees to adjust schedules. Compared with traditional employees and self-employed people, platform workers report a higher degree of work–family conflict, and off-hours work contact can only explain some of the differences (Glavin et al., 2024). In the hotel context, employees’ daily AI awareness may aggravate work–family conflict by weakening psychological detachment, while weekly ICT availability requirements may make employees continuously connected with work outside formal working hours, thus damaging their family-role performance (Yi and Kumar, 2026; Zhang et al., 2026). The direct path is consistent with the above conclusion: even if employees can still make certain adjustments to individual shifts, algorithmic scheduling may encroach on family time through work-time fluctuations, undesirable shifts, schedule notifications or the expectation of availability. However, since these specific temporal intrusion mechanisms were not measured separately in this study, the path coefficient should be interpreted as evidence that low work-time control is not enough to fully explain the relationship between algorithmic scheduling exposure and work–family conflict.

The structural relationship between work–family conflict and work-related burnout is the strongest in the model, and the serial indirect relationship between algorithmic scheduling exposure and work-related burnout through low work-time control and work–family conflict also reaches a significant level. This result is consistent with the meta-analysis evidence. Existing studies have shown that the interference of work with family is closely related to work-related and general strain outcomes (Amstad et al., 2011). Hospitality research also found that there was a significant correlation between work–non-work conflict and burnout (Xu and Cao, 2019). In addition, the results also echo hotel and service research, which shows that work–family conflict can predict emotional exhaustion and transmit the impact of workplace demands to employees’ functional performance (Karatepe, 2013; Wang et al., 2021). Relatively recent technology research also found that AI awareness will form a serial association with emotional exhaustion through job insecurity and work interference with family (Zheng and Zhang, 2025). The added value of this research model is that it identifies a different starting point that is closer to the daily work experience of employees: it is not employees’ worry about being replaced by technology in the future, but the algorithmic management function of directly allocating employees’ working time.

Finally, higher FSSB was associated with a weaker exposure–low-control relationship and a smaller serial indirect association, including in the adjusted model. This finding is consistent with the relevant evidence of the hospitality industry, that is, FSSB can improve work–family balance and promote more positive family outcomes and service outcomes (Ye et al., 2024). At the same time, this result is also consistent with an emerging view, that is, in hotels that implement algorithmic management, front-line managers undertake translation work, and need to interpret, adjust and override the system output when necessary (Jianu et al., 2025a). It is worth noting that even at a high level of FSSB, the positive relationship between algorithmic scheduling exposure and low work-time control is still significant. Thus, higher FSSB was associated with a weaker, but still significant, exposure–control relationship. This pattern is compatible with a supportive role for supervisors. It does not establish that they restored negotiability or overcame rigid system rules, because those mechanisms and actual override authority were not independently verified. The descriptive reports of supervisory scheduling permissions do not establish how those permissions were used. Moreover, without a separate general-support measure, the interaction cannot establish that the association is uniquely attributable to family-specific support rather than a broader supportive supervisory climate.

The Chinese context may help contextualize the buffering association of FSSB. Research in Chinese hotels links supervisor–subordinate guanxi with perceived autonomy support (Zhang et al., 2023), while earlier workplace research documents reliance on informal, case-by-case work–family accommodation (Xiao and Cooke, 2012). Supervisory support may therefore be particularly consequential where accommodation depends on managerial discretion. Its role could differ in settings with more formalized working-time protections and family-support arrangements. However, neither guanxi nor supervisors’ actual scheduling authority was measured, so these contextual explanations remain tentative.

### 5.1 Theoretical contributions

This study mainly makes three theoretical contributions. First, it no longer regards algorithmic management as a single technology exposure, but divides it into several specific management functions with different functional logics, thus advancing hospitality technology research. AI awareness research mainly explains how employees respond to the potential possibility of being replaced by technology, while algorithmic scheduling is a management practice that has been actually implemented, which directly determines when employees work. This study reveals the relationship between this specific function, work-time control and work interference with family, so as to explain why the specific content of algorithmic decision-making is crucial. Algorithmic management affects employees not only because of its technological novelty, but also because different algorithmic functions will reconstruct different types of work resources.

Second, this study extends the work–home resources model to the algorithmic temporal governance context. The results reveal a sequence of action with theoretical order: algorithmic scheduling constitutes a contextual work demand, low work-time control reflects the loss of contextual work resources, work–family conflict reflects cross-domain interference, and work-related burnout represents cumulative work-related strain (ten Brummelhuis and Bakker, 2012). Distinguishing management practice from the resource experience of employees can avoid the simple combination of technological arrangement and work design into a general adverse working condition. The complementary partial mediation result further refines this resource explanation: although work-time control plays an important role, it is not the only temporal path for algorithmic scheduling to enter employees’ family life.

Third, the moderating effect conceptualizes hotel scheduling as hybrid temporal governance. Existing FSSB studies generally regard supervisors as a support resource to help employees cope with work–family requirements. In this study, the role of FSSB occurs earlier, that is, at the interface where the time allocation generated by the system is transformed into employees’ actual scheduling experience. FSSB weakens the relationship between algorithmic scheduling and the loss of work-time control, which shows that human supervision is still a constituent element of algorithmic management, and not only provides remedial support after negative consequences have occurred. This result is consistent with a sociotechnical interpretation in which supportive supervision conditions the association between automated scheduling and experienced work-time control. The significant moderated serial mediation effect extends this conditional association across work and family domains, but does not directly demonstrate that supervisors explained, challenged, or adjusted algorithmic allocations. Its incremental family-specific contribution beyond general support remains untested.

### 5.2 Practical implications

Hotels should not only evaluate the scheduling system based on forecast accuracy, labor cost or personnel coverage, but also pay attention to the quality of working time formed by the system. In the process of system procurement and design, employees’ availability and personal preferences should be fully included, sufficient advance notice should be provided, avoidable short-notice shift changes should be reduced, and the scheduling rules should be easy to understand. The hotel should also provide clear and visible scheduling adjustment and appeal channels for employees, so that they can make modification requests or raise objections to time allocation without punishment. In addition to operational efficiency, the hotel can also include indicators such as the fluctuation of working hours per week, the advance time of scheduling notice, the number of rejected leave applications, scheduling contact during non-working hours and the manual adjustment rate into daily monitoring.

The supervisor should not only have the ability of family support, but also have the actual authority of scheduling adjustment. Relevant training should combine FSSB with algorithmic literacy, so that managers can explain algorithm output, assist employees to change shifts, adjust unreasonable time arrangements, and feed back recurrent problems to system designers. Simple emotional support cannot make rigid scheduling negotiable. Therefore, the hotel should clearly specify the circumstances under which the manager can override or adjust the system arrangement, record the corresponding reasons, and prevent the manager from simply shifting the responsibility to the algorithm” At the same time, the hotel should also set up protected non-working hours, and limit scheduling notices and availability requirements during non-working hours. There is still a direct relationship between algorithmic scheduling and work–family conflict, which indicates that only improving employees’ work-time control may not be enough to completely eliminate temporal intrusion. Because higher-level FSSB can only weaken but cannot completely eliminate this relationship, supportive supervision should be used as a supplement to employee-centered system design, and cannot replace it.

### 5.3 Limitations and future research

There are still some limitations in this study. First, the single-wave, self-reported research design cannot establish the temporal sequence or causal relationship between variables. Future research can adopt multi-wave survey or field experiment design to distinguish algorithmic scheduling exposure, the work-time control felt by employees, family interference and work-related burnout in terms of time. Voluntary, privacy-protected wearable or smart-device data could complement surveys and scheduling logs by capturing movement, posture, and activity–recovery periods. Repeated assessments could relate these indicators to stress, job satisfaction, perceived work-time control, and work–family conflict, without treating device outputs as direct measures of psychological experience. Second, the scale of algorithmic scheduling exposure only measures perceived automation intensity, not transparency, fairness, employee input, negotiability, or objective scheduling outcomes. This measurement boundary limits the interpretation of H1: the positive exposure–low-control association cannot be attributed to opacity or rigidity, or assumed to apply equally across different system designs. Unmeasured process quality and organizational practices may help explain the observed association and the present data cannot distinguish these explanations. The FSSB interaction likewise does not directly establish improved negotiability or actual supervisory override. Future research can combine employee reports with scheduling logs, and, respectively, test different mechanisms such as scheduling predictability, undesirable working hours, non-working-hours availability requirements, algorithmic opacity and fairness. Finally, based on the sample of front-line hotel employees in China, the research conclusion still needs to be further verified in different countries and service industries. Purposive recruitment through participating hotels in Shandong also permits selection at both hotel and employee levels. Hotels open to research may differ in scheduling practices or supervisory climate, while employees reached by hotel contacts or willing to respond may differ in workload or scheduling experiences. Anonymity and the reported usable completion rate cannot rule out these biases. Selection may affect the magnitude or direction of the estimated associations as well as their generalizability, and its extent cannot be established from this survey. Replication across regions and seasons, with documented hotel and employee participation and comparisons with nonparticipants where possible, is needed. In the future, multilevel matched data can be used to investigate the authority of scheduling adjustment actually owned by the supervisor, and to test whether FSSB has a stronger impact on the negotiability of scheduling than emotional support.

The adjusted model addresses only the measured working-time and family circumstances. Separate overtime, night-work frequency, general supervisor or organizational support, job demands, general autonomy, and negative affectivity were not measured. Standard background characteristics were collected but were not included in this focused adjustment; residual demographic, departmental, and employment-related confounding remains possible. Fatigue or negative affect may influence all self-reports, including exposure, and the CMV diagnostics cannot exclude this possibility. These boundaries prevent causal attribution and a claim that FSSB contributes beyond general support. Future studies should measure the competing constructs directly and establish discriminant validity before comparing their contributions. Furthermore, Respondents came from 12 hotels, but hotel identifiers were not retained at the respondent level and cannot be reconstructed. Hotel fixed effects, intraclass correlations, cluster-adjusted standard errors, and multilevel estimation could therefore not be implemented. If respondents within a hotel share unmodeled influences, the individual-level bootstrap intervals may be too narrow and hotel-level confounding may remain. Accordingly, significance and the moderated indirect association require confirmation in samples with identifiable hotel clusters.

## Data Availability

The raw data supporting the conclusions of this article will be made available by the authors, without undue reservation.
