# Supplementary material for: When algorithms set the roster: algorithmic scheduling, work-time control, and work-related burnout among hotel employees
Source: Front Psychol. 2026 Oct 7;17:1958975. doi: 10.3389/fpsyg.2026.1958975 (PMC13683223; doi:10.3389/fpsyg.2026.1958975)
Supplement: Supplementary file 1 [file Table_1.DOCX]

**Appendix 1. Complete measurement scales**

| **Construct** | **Code** | **Item wording** | **Source** |
| --- | --- | --- | --- |
| **Algorithmic scheduling exposure** | SCH1 | An automated system decides when I work and when I do not. | Parent-Rocheleau et al. (2024) |
|  | SCH2 | My work schedule is made by an automated system. |  |
|  | SCH3 | An automated system is responsible for determining my working hours. |  |
|  | SCH4 | My working hours are determined automatically by an electronic system. |  |
| **Low work-time control** | LTC1 | Unpredictable hours from week to week | McNamara et al. (2011) |
|  | LTC2 | Short notice about shift changes |  |
|  | LTC3 | Not getting time off requested |  |
| **Family-supportive supervisor behaviors** | FSSB1 | Your supervisor makes you feel comfortable talking to him/her about your conflicts between work and non-work. | Hammer et al. (2013) |
|  | FSSB2 | Your supervisor demonstrates effective behaviors in how to juggle work and non-work issues. |  |
|  | FSSB3 | Your supervisor works effectively with employees to creatively solve conflicts between work and non-work. |  |
|  | FSSB4 | Your supervisor organizes the work in your department or unit to jointly benefit employees and the company. |  |
| **Work-family conflict** | WFC1 | The demands of my work interfere with my home and family life. | Netemeyer et al. (1996) |
|  | WFC2 | The amount of time my job takes up makes it difficult to fulfill family responsibilities. |  |
|  | WFC3 | Things I want to do at home do not get done because of the demands my job puts on me. |  |
|  | WFC4 | My job produces strain that makes it difficult to fulfill family duties. |  |
|  | WFC5 | Due to work-related duties, I have to make changes to my plans for family activities. |  |
| **Work-related burnout** | WRB1 | Do you feel worn out at the end of the working day? | Kristensen et al. (2005) |
|  | WRB2 | Are you exhausted in the morning at the thought of another day at work? |  |
|  | WRB3 | Do you feel that every working hour is tiring for you? |  |
|  | WRB4 | Do you have enough energy for family and friends during leisure time? |  |
|  | WRB5 | Is your work emotionally exhausting? |  |
|  | WRB6 | Does your work frustrate you? |  |
|  | WRB7 | Do you feel burnt out because of your work? |  |
